# Phosphate Adsorption from Membrane Bioreactor Effluent Using Dowex 21K XLT and Recovery as Struvite and Hydroxyapatite

**DOI:** 10.3390/ijerph13030277

**Published:** 2016-03-03

**Authors:** Tanjina Nur, Paripurnanda Loganathan, Jaya Kandasamy, Saravanamuthu Vigneswaran

**Affiliations:** Centre for Technology in Water and Wastewater, Faculty of Engineering and Information Technology, University of Technology Sydney, Broadway, Sydney NSW 2007, Australia; Tanjina.Nur@uts.edu.au (T.N.); pari.loganathan@yahoo.com (P.L.); jaya.kandasamy@uts.edu.au (J.K.)

**Keywords:** phosphate, adsorption, struvite, hydroxyapatite, wastewater, fertiliser

## Abstract

Discharging phosphate through wastewaters into waterways poses a danger to the natural environment due to the serious risks of eutrophication and health of aquatic organisms. However, this phosphate, if economically recovered, can partly overcome the anticipated future scarcity of phosphorus (P) resulting from exhaustion of natural phosphate rock reserves. An experiment was conducted to determine the efficiency of removing phosphate from a membrane bioreactor effluent (pH 7.0–7.5, 20, 35 mg phosphate/L) produced in a water reclamation plant by adsorption onto Dowex 21K XLT ion exchange resin and recover the phosphate as fertilisers. The data satisfactorily fitted to Langmuir adsorption isotherm with a maximum adsorption capacity of 38.6 mg·P/g. The adsorbed phosphate was quantitatively desorbed by leaching the column with 0.1 M NaCl solution. The desorbed phosphate was recovered as struvite when ammonium and magnesium were added at the molar ratio of phosphate, ammonium and magnesium of 1:1:1 at pH 9.5. Phosphate was also recovered from the desorbed solution as hydroxyapatite precipitate by adding calcium hydroxide to the solution at a phosphate to calcium molar ratio of 1:2 at pH 7.0. The P contents of struvite and hydroxyapatite produced were close to those of the respective commercial phosphate fertilisers.

## 1. Introduction

In recent decades the wastewater treatment industry has identified the discharge of phosphorus (P) from industries, agriculture and municipal effluents into waterways as a risk to natural environments due to the serious effects of eutrophication and health of aquatic organisms. Eutrophication is the abundance of aquatic plants, growth of algae, and depletion of dissolved oxygen [1,2]. In cases of severe eutrophication, dissolved oxygen gets depleted causing fish death when algae decay [3,4]. The algal biomass produced by eutrophication can also hinder water treatment by blocking filters or penetrate through them causing bad odour and taste to the treated water [5]. The blue-green algae produced can release compounds that can be toxic to fish and other aquatic organisms [6]. These conditions are also of potential risk to human health, if shellfish contaminated with algal toxins is consumed by humans [7].

As the global supplies of clean water diminish and people’s demand for water rises, advanced wastewater treatment is becoming an international focus for the rational use of scarce water resources and as a means of safeguarding aquatic environments from the damage caused by wastewater disposal. In recent times the treatment of domestic urban wastewater using the membrane bio-reactor (MBR) process has become popular as effluent standards are more stringent and current water conservation measures strongly emphasise water reuse applications. The MBR system consists of a suspended-growth biological reactor combined with a membrane unit process [8]. MBR is a promising process in wastewater treatment for its ability in completely removing solids, superior removal of nutrients and organic matter, high loading rate capabilities, low/zero sludge production and small footprint. This makes the MBR particularly suitable for water reuse. Although the MBR process can remove most organic matter and nutrients from water, it fails to remove P when exposed to peak and variable loads depending on the operating conditions [9].

Phosphate is an important resource used in agriculture and many other industries. However, P resources are limited and there have been some alarming reports that deposits of high-grade P ores are likely to be depleted within the next few decades [10,11]. Phosphate has no substitute in food production and in a world of 9 billion people by 2050, securing sufficient P will be critical for future food security. Yet the World’s main source of P—phosphate rock—is non-renewable and becoming increasingly scarce and expensive. Peak P was estimated to occur by 2035, after which the demand would outstrip the supply [11]. As P use efficiency is low, only 15%–20% of applied P is used by crops and animals, and the remaining P is shunted into various waste streams [12]. 

The P in these waste streams, if economically recovered, can contribute to a sustainable management of P resources [12]. Therefore, the recovery of P from P-containing wastewater is essential for developing an alternative source that can help overcome the global challenge of P scarcity. To illustrate this point, a P recovery scenario in Australia can be considered. The daily amount of wastewater generated in Sydney, Australia is over 1200 ML. Assuming a typical raw sewage P concentration of 11 mg/L, Sydney’s wastewater system generates 13.2 tonnes of P on a daily basis or 4820 tonnes of P annually. 

Phosphorus is present in wastewater as orthophosphates, condensed phosphates and various organic phosphate fractions. In the MBR system only a small amount of P is used for cell metabolism and growth (1%–2% of the total suspended solids (TSS) mass in the mixed liquor) [13]. Since significant amounts of P cannot be removed in MBR treatment, another post-treatment strategy is required along with MBR for P removal. Phosphorus can also be removed through enhanced biological phosphorus removal (EBPR), however, the successful operation of EBPR depends on many process operational factors, especially variations in wastewater quality [14]. Furthermore, one cannot remove the P below a particular concentration through biological processes. Of all the methods of P removal, adsorption/ion exchange strategies are promising because they are simple and economical, result in less sludge production and therefore experience minimal disposal problems [15]. 

The ion exchange method provides a more selective means of separating ions from solution. In fact the ion exchange process using selective ion exchange materials is ideal for reducing phosphate to near-zero levels provided that the ion exchange resin is phosphate selective, cost-effective and amenable to efficient regeneration and reuse [16]. Different ion exchange resins have been employed to remove P from wastewater such as Purolite A500P, Purolite A520E, Purolite FerrIX A33E, Amberlite IRA910Cl and Amberjet 1200Na [17,18,19]. Ion exchange resins are used as filter media in filter-based systems and after a period of usage they become saturated with P, leading to a decline in their removal efficiency. The exhausted resins can be regenerated using different regeneration reagents or a mixture of regeneration reagents such as NaCl, NaOH, CaCl_2_, HCl, and Na_2_CO_3_ [15]. Phosphorus so removed can be recovered by precipitation with calcium/or magnesium salts and employed as phosphorus fertiliser [20,21]. Alternatively, it can be diluted with irrigation water for fertilising irrigated crops [15]. Very few studies have been reported on this method of P recovery from real wastewater or MBR treated water although such studies have been conducted on synthetic water [18]. Tsuji *et al.* [21] used a phosphate adsorbent to recover P from municipal wastewater and successfully produced calcium phosphate fertiliser by adding calcium chloride to the solution containing the recovered P.

In this research, MBR effluent from a water reclamation plant in Gordon, Sydney, Australia was used for the removal and recovery of P using Dowex 21K XLT ion exchange resin. The basis for selecting Dowex 21K XLT resin is that it is a strong base resin and in our previous study, we found that it had a high adsorption capacity for phosphate [22]. This resin was also used to selectively adsorb gold–cyanide complex in a previous study [23]. 

The objectives of this study were to: (i) determine the adsorption capacity of Dowex 21K XLT for P from the MBR treated water using batch and column modes of adsorption experiments; (ii) investigate the feasibility of struvite and hydroxyapatite production from the desorbed P and (iii) characterise the struvite and hydroxyapatite products using chemical analysis. Nitrate and sulphate concentrations in the MBR treated water were also high as phosphate concentration but no attempt was made to recover them as fertilisers because no shortage of fertilisers containing these nutrients is expected in the near future unlike in the case of phosphate fertilisers. The novel feature of the study is the removal of phosphate from wastewater containing a range of phosphate-competitive co-ions under dynamic condition. This is followed by an experimental investigation on desorption and recovery of phosphate as fertilisers for beneficial use. The study conducted on MBR treated water at the Gordon plant is expected to help other plants in Australia and other countries recover P from wastewaters and produce beneficial P fertilisers.

## 2. Material and Methods

### 2.1. Feed Water

MBR effluent from a water reclamation plant in Gordon, Sydney, Australia was used as feed water in this research. In this plant, a sewer mining system utilising MBR technology served to transform municipal wastewater into a usable source of irrigation water for greens and fairways. This plant consisted of screening, two-stage biological reactor (anoxic and aerobic zones), ultrafilter and ultraviolet disinfection. The two-stage biological reactor followed by ultrafiltration is called MBR. The ultrafiltration used ZeeWeed 500 membrane produced by General Electric (GE, Schenectady, NY, USA). ZeeWeed 500 membranes were reinforced hollow-fibres that had been widely used in wastewater treatment for water reuse. By implementing the GE MBR system, the plant was able to recover 98% of its wastewater. This plant was designed to treat an inflow rate of 300 m^3^/day, with the daily activated sludge waste volume close to 5 m^3^/day. Treated water of close to 110 ML/year is being produced at this plant. The characteristics of the MBR treated water are given in Table 1. A recovery of 90% of phosphate could lead to 3.85 metric tonnes/ year of phosphate.

### 2.2. Ion Exchange Resin

Dowex 21K XLT ion exchange resin was used in the study. It is a strong base Styrene-DVB matrix anion exchange resin obtained from Dow Chemical Pte Ltd. (Midland, MI, USA) [24]. The quaternary methylamine functional group with chloride as the counter ion in the resin gives the resin the ability to adsorb other anions through the ion exchange process. The physical state of the resin consisted of yellow spherical beads 0.3–1.2 mm in diameter with a density of approximately 1.08 g/mL.

### 2.3. Chemical Analysis

The analyses of nitrate, phosphate and sulphate were carried out using a model 790 Personal IC ion chromatograph (Herisau, Switzerland) equipped with an auto sampler and conductivity cell detector. The separation of anions was achieved using an A SUPP column 3 (150 mm × 4 mm). Na_2_CO_3_ (3.2 mmol/L) and NaHCO_3_ (1.0 mmol/L) were used as mobile phase with a flow rate of 0.9 mL/min. The concentrations of calcium and magnesium were measured by employing a Microwave Plasma-Atomic Emission Spectrometer (Agilent Technologies, Santa Clara, CA, USA). Ammonium concentration was measured by the cell test method (Spectroquant, Merck, Darmstadt, Hesse, Germany) using a photometer (NOVA 60, Merck). The pH was measured using a HQ40d portable pH Meter (Hach, Loveland, CO, USA).

### 2.4. Characteristics of Dowex 21K XLT

Energy dispersion X-ray spectroscopy (EDS) analysis was conducted using a scanning electron microscope (Supra 55VP Field Emission, Zeiss, Oberkochen, Germany) in conjunction with energy dispersion spectrometer operated at 15 kV to determine the elemental composition of Dowex 21K XLT. The Dowex sample was fixed onto a stub sample holder using a carbon tape. Zeta potential measurements were conducted to determine the surface charge characteristics of the Dowex 21K XLT. The Dowex was ground to a smaller size so that they could be suspended in water. 0.1 mg of this material was added to 100 mL Milli-Q water in different flasks and pH adjusted to 4–8 using diluted HCl and NaOH solutions. The flasks were agitated at a speed of 120 rpm and then zeta potential was measured using a Zetasizer Nano instrument (Nano ZS Zen 3600, Malvern, Worcestershire, UK). 

### 2.5. Effect of pH

The effect of pH on phosphate adsorption was studied by agitating 1 g of Dowex 21K XLT at pHs from 3 to 10 with 100 mL of MBR treated water in a set of glass flasks. The suspensions were agitated in a flat shaker at a shaking speed of 120 rpm for 24 h at room temperature (24 ± 1 °C). Aqueous samples were taken and the concentrations of P were measured.

### 2.6. Batch Mode Adsorption Experiment

Equilibrium adsorption experiments were conducted in a set of glass flasks with 100 mL solutions of MBR treated water and adsorbent doses of 0.1–10 g/L at room temperature (24 ± 1 °C) and pH 7.0–7.5. The suspensions were agitated for 24 h in a flat shaker at a shaking speed of 120 rpm. Experiments were duplicated and the average values were taken for data analysis. The differences between duplicate values were within ±2%. The amounts of nitrate, phosphate and sulphate adsorption at equilibrium, q_e_ (mg/g), were calculated using Equation (1):

(1) $$q_{e}=\frac{\left(C_{0}-C_{e}\right)V}{M}$$

where, C_0_ = initial concentration of nitrate, phosphate and sulphate (mg/L); C_e_ = equilibrium concentration of the nitrate, phosphate and sulphate (mg/L); V = volume of the solution (L) and M = mass of adsorbent (g). The adsorption data were treated with Langmuir adsorption isotherm (Equation (2)):

(2) $$q_{e}=\frac{q_{\text{max}}K_{L}C_{e}}{1+K_{L}C_{e}}$$

where, C_e_ = equilibrium concentration of nitrate, phosphate and sulphate (mg/L), q_e_ = amount of nitrate, phosphate and sulphate adsorbed per unit mass of Dowex 21K XLT (mg/g), q_max_ = maximum amount of nitrate, phosphate and sulphate adsorbed per unit mass of Dowex 21K XLT (mg/g); K_L_ = Langmuir constant related to the energy of adsorption (L/mg).

### 2.7. Column Mode Adsorption Experiment

The fixed-bed column used comprised a 2.5 cm inner diameter pyrex glass tube. At the bottom of the column, a stainless steel sieve was attached followed by a layer of glass beads in order to provide a uniform flow of the solution through the column. A known quantity (56 g) of the resin was packed in the column to yield the desired bed height (12 cm). MBR treated water solutions were pumped upward through the column at a desired filtration velocity (2.5 m/h) controlled by a peristaltic pump. The effluents at the outlet of the column were collected at regular time intervals and the major anions present in MBR treated water such as nitrate, phosphate and sulphate concentrations were measured. At the pH of 7.0–7.5 of the MBR treated water there could have been appreciable concentrations of carbonate and bicarbonate in the influent solutions due to absorption of CO_2_ from air but the removal of these anions was not studied.

The breakthrough curves show the loading behaviour of phosphate to be removed from solution in a fixed-bed column. These are usually expressed in terms of adsorbed nitrate, phosphate and sulphate concentrations (C_ad_), inlet nitrate, phosphate and sulphate concentrations (C_0_), outlet nitrate, phosphate and sulphate concentrations (C_t_) or normalised concentrations defined as the ratio of outlet nitrate, phosphate and sulphate concentrations to inlet nitrate, phosphate and sulphate concentrations (C_t_/C_0_) as a function of time. The maximum column adsorption capacity, q_total_ (mg/g) is equal to the area under the plot of the adsorbed phosphate-P concentration, C_ad_ (C_ad_ = C_0_ − C_t_) (mg/L) *versus* effluent collection time (t, min) and was calculated from Equation (3):

(3) $$q_{\text{total}}=\frac{Q}{1000}\int_{t=0}^{t=\text{total}}C_{\text{ad}}\;\text{dt}$$

Equilibrium uptake q_eq_ (mg/g) or maximum adsorption capacity of the column is defined by Equation (4) as the total amount of adsorbed nitrate, phosphate and sulphate concentrations (q_total_) per g of adsorbent (M) at the end of the total flow time:

(4) $$q_{\text{eq}}=\frac{q_{\text{total}}}{M}$$

### 2.8. Desorption of Phosphate

Desorption of the adsorbed phosphate was carried out by leaching the columns with 0.1 M NaCl. The leachates were collected after 30 min and 60 min and the concentration of phosphate was measured. Desorption of sulphate and nitrate was not measured because the recovery of these nutrients is not as important as the recovery of phosphate which is the nutrient perceived to be of future scarecity.

### 2.9. Struvite Precipitation

Phosphate was recovered as struvite (MgNH_4_PO_4_∙6H_2_O) from the regenerated solution by adding 0.284 g magnesium chloride (MgCl_2∙_6H_2_O) and 0.092 g ammonium sulphate ((NH_4_)_2_SO_4_) to 200 mL of the leachate collected at 30 min to provide a phosphate, ammonium and magnesium molar ratio of 1:1:1 and the solution pH adjusted to 9.5. For the leachate collected at 60 min, 0.163 g magnesium chloride and 0.053 g ammonium sulphate were added and the solution pH adjusted as before. Previous studies reported that the above molar ratio and pH between 8.5 and 9.5 were the optimum conditions for producing struvite [25,26]. The experiments were conducted in a set of glass flasks containing 200 mL of solution. The solutions were agitated in a flat shaker at a shaking speed of 150 rpm for 3 h at room temperature (24 ± 1 °C). During this process the pH of the samples was adjusted to the desired value of 9.5 by adding sodium hydroxide (NaOH). When the reaction time had elapsed the precipitate that had formed was collected by filtration through a 0.2 mm membrane filter. The precipitates were kept in an oven to dry at a temperature of 40 °C for 24 h and the weights were measured. 

### 2.10. Hydroxyapatite Precipitation

A portion of the remaining desorbed solutions collected at 30 min and 60 min (200 mL each time) were used to recover P as hydroxyapatite by adding calcium hydroxide (Ca(OH)_2_) to provide phosphate to calcium molar ratios of 1:0.5 and 1:2 and pH adjusted to 7.0. The weights of Ca(OH)_2_ added for the ratio of 1:0.5 were 0.052 g and 0.028 g for the solutions collected at 30 min and 60 min, respectively. Similarly the appropriate weights of Ca(OH)_2_ were used for the 1:2 molar ratio. Our previous study showed that the phosphate and calcium contents in the precipitate increased gradually in tandem with an increase in the molar ratio and they were highest at 1:0.5 and 1:2 ratio [22]. The experiments were conducted in a set of glass flasks containing 200 mL of desorbed solution. The solutions were agitated in a flat shaker at a shaking speed of 150 rpm for 3 h at room temperature (24 ± 1 °C). The precipitates formed were collected by filtration through a 0.2 mm membrane filter and dried at room temperature for 24 h. 

### 2.11. Phosphorus Content in Struvite and Hydroxyapatite

Phosphorus content in struvite and hydroxyapatite was measured by placing 0.01–0.2 g of dried precipitate samples in a set of beakers containing 100 mL of 1% (w/w) nitric acid solution and agitating the suspension using a magnetic stirrer for 30 min at room temperature (24 ± 1 °C). The suspensions were filtered through a 0.2 mm membrane filter and the concentration of phosphate in the filtrate was measured.

## 3. Results and Discussion

### 3.1. EDS Analysis

**Figure 1:**
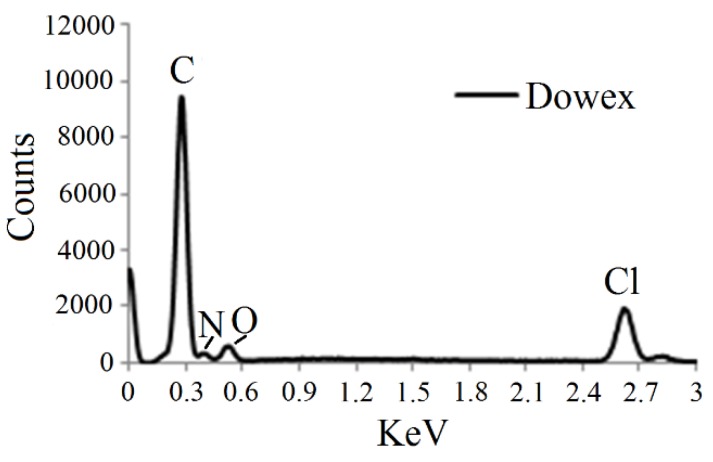
EDS analysis of Dowex 21K XLT.

EDS analysis revealed that the elemental composition of Dowex 21K XLT consists of C, O, N, and Cl (Figure 1). Some of the C in the EDS spectra would have come from the carbon tape used to fix the sample onto the stub sample holder. While C, O, and N formed the styrene-DVB matrix of Dowex, Cl is the exchangeable anion adsorbed to the positively charged quarternary methyl amine functional group [23,24]. The EDS data showed that the percentage of Cl was 11.7 which was approximately the same as 10.1% reported for this resin by Ok and Jeon [23].

### 3.2. Zeta Potential and Effect of pH on P Adsorption

**Figure 2:**
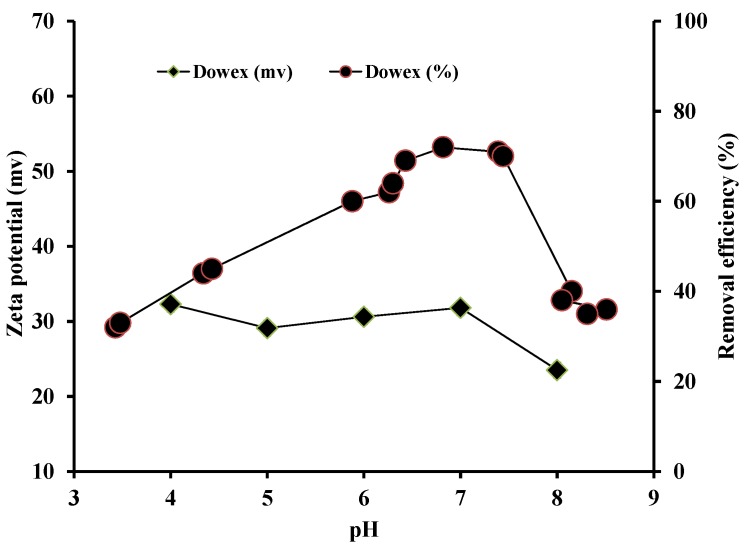
Effect of pH on zeta potential and phosphate removal efficiency of Dowex 21K XLT.

Zeta potential is the electrical potential at the boundary of the hydrodynamic shear plane of the charged adsorbents close to an adsorbent surface where adsorption of ions from solution phase occurs and it is positively related to the surface charge. The higher the positive zeta potential the higher the anion exchange capacity, resulting in adsorption of larger amounts of anions such as phosphate. The positive zeta potential values obtained on the Dowex samples were nearly the same for the pH values 4 to 7 and they were slightly reduced at pH 8 (Figure 2). The reduction in zeta potential at the highest pH of 8 was due to the deprotonation of quaternary methylamine functional groups of Dowex at this pH resulting in decreased number of positive charges.

The constant zeta potential in the pH range 4 to 7 suggests that the adsorption of phosphate should be the same in this pH range. However, the adsorption data shows that phosphate adsorption increased from pH 4 to 7 (Figure 2). This discrepancy may be due to the excess amounts of Cl added in HCl to reduce the pH in the experiment. Cl may have competed with phosphate for adsorption resulting in decline of phosphate adsorption as pH decreased. At pHs beyond pH 7.5 the phosphate adsorption again decreased. This is because of decrease in the positive surface charges as indicated by the reduction in zeta potential (Figure 2).

### 3.3. Batch Adsorption Experiment

**Figure 3:**
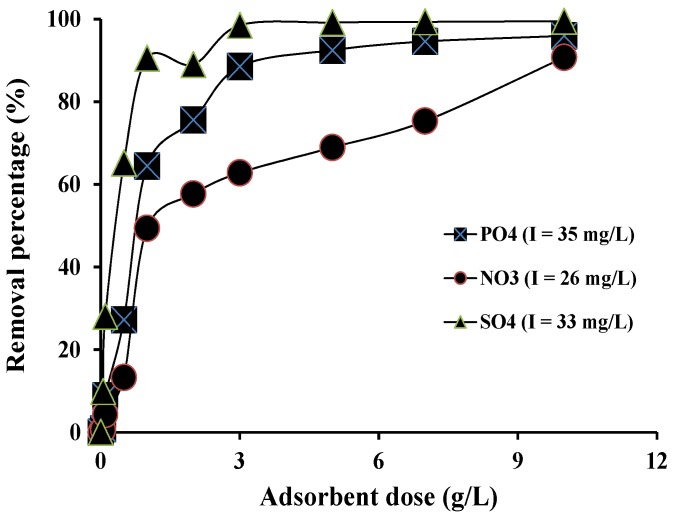
Effect of Dowex 21K XLT resin dose on the percentage removal of phosphate, nitrate and sulphate from MBR treated solution (I is initial concentration).

The percentage removal of all anions increased as the adsorbent dosage was increased due to the availability of more adsorption sites (Figure 3). The removal efficiencies were 99%, 90% and 60% for sulphate, phosphate and nitrate, respectively at an adsorbent dose of 3 g/L. The corresponding values of removal efficiencies at an adsorbent dose of 10 g/L were 99%, 96% and 91%, respectively. The reason for the higher adsorption of sulphate than the other anions was that sulphate has two negative charges whereas phosphate has between one and two charges (H_2_PO_4_^−^, HPO_4_^2−^) and nitrate has only one charge at pH 7.0–7.5 of the MBR water. Increased number of negative charge of the anion is expected to increase the electrostatic adsorption of the anions on the positively charged Dowex 21K XLT ion exchanger.

### 3.4. Batch Adsorption Capacity

The adsorption data satisfactorily fitted to the Langmuir adsorption isotherm (R^2^ = 0.80 − 0.83). The Langmuir maximum adsorption capacities (mg/g) calculated from the isotherms for sulphate, phosphate, and nitrate were 86.9, 38.6, and 11.1, respectively. The adsorption capacity for phosphate of 39 mg/g was among the highest values reported in the literature for most ion exchangers [15]. For example, Marshall and Wartelle [27] developed an anion exchange resin from soybean hulls consisting of lignocellulose by chemical modification with an organic nitrogen compound. The authors found that this resin had a Langmuir adsorption capacity of 19.5 mg/g at pH 7.0. They compared this value with values reported for a commercial cellulose-based resin of 14.3 mg /g and for a commercial synthetic resin, Amberlite IRA-400 which had a value of 32.2 mg/g. Hamoudi *et al.* [28] produced an ammonium functionalised mesoporous silica compound called MCM-48 and reported that it had a maximum adsorption capacity of 15.5 mg/g at pH 6.0. Gupta *et al.* [29] discovered that Purolite A500P anion exchange resin had a Langmuir adsorption capacity of 21 mg/g for phosphate at pH 7.5. These literature values were obtained from experiments conducted on synthetic waters containing only phosphate, whereas the current study was conducted using MBR treated water containing other anions as well. It is interesting to note that despite the competition from other anions in the solution the phosphate adsorption capacity was higher than the above literature values.

### 3.5. Fixed-Bed Column Adsorption Studies

**Figure 4:**
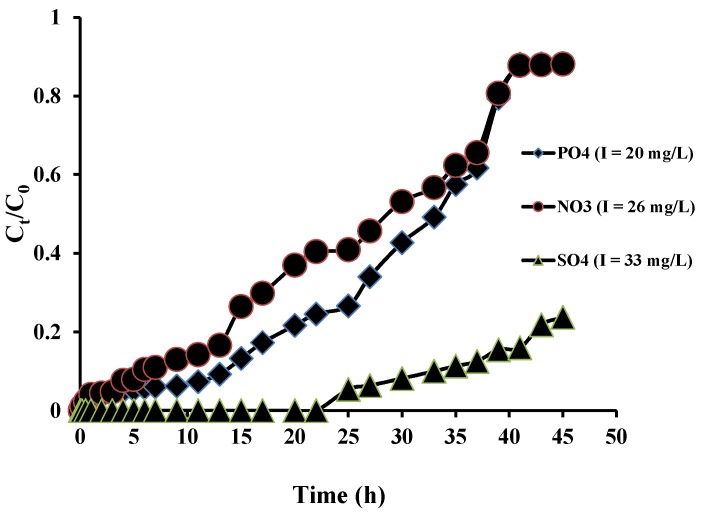
Breakthrough curves for Dowex 21K XLT resin (I is influent concentration).

The breakthrough curve obtained for the Dowex 21K XLT was similar for nitrate and phosphate and the column was saturated after 45 h (Figure 4). However, for sulphate it was not saturated as the resin had a higher adsorption capacity for this anion compared to phosphate and nitrate. The breakthrough adsorption capacities for nitrate, phosphate and sulphate calculated from the breakthrough curves were 13.3, 14.9 and 29.7 mg/g, respectively. The order of the adsorption capacities obtained in the column experiment is the same as that in the batch experiment (sulphate > phosphate > nitrate).

### 3.6. Desorption of Phosphate

Desorption of phosphate adsorbed to the resin was carried out by leaching the column using 0.1 M NaCl at a flow rate of 10 m/h for 60 min. Approximately 70% of the adsorbed phosphate was desorbed within the first 30 min (42 bed volumes) and the remaining 30% was desorbed within the next 30 min. The phosphate concentrations in the two desorbed solutions were 0.007 M and 0.004 M, respectively. A total volume of 1.2 L of leachate was collected.

### 3.7. Phosphate Recovery as Struvite 

The concentrations of phosphate, ammonium and magnesium in the precipitate are presented in Table 2. The values for these anions found in the precipitate from the desorbed solution collected during the first 30 min were 41.7%, 10.9%, and 12.6%, respectively. These values closely matched the theoretical composition of struvite, *i.e.*, phosphate, ammonium and magnesium of 38.8%, 7.3%, and 9.8%, respectively [26]. The slightly lower values obtained for the precipitate may be due to other ions contributed by the desorbing agent NaCl and ions that were originally present in the MBR effluent. The respective concentrations were smaller in the struvite precipitate formed in the solution collected in the last 30 min. This may have probably been due to the smaller concentration of phosphate in the desorbed solution.

### 3.8. Phosphate Recovery as Hydroxyapatite 

The compositions of hydroxyapatite at the two molar ratios of phosphate to calcium used in the experiment are presented in Table 3. The phosphate contents of the precipitates formed from the desorbed solution collected during the first 30 min were 25.1% and 38.5% for the phosphate to calcium ratios 1:0.5 and 1:2, respectively, while the corresponding calcium contents were 35.1% and 32.8% (Table 3). The corresponding P contents were 8.2% and 12.6%, respectively, which are within the range of values reported in the literature for natural phosphate rocks (8.9%–17.2%) [30]. The P content of 12.6% obtained for the higher phosphate to calcium ratio (1:2) matches well with the P content of 13.1% reported by Tsuji *et al.* [21] for a hydroxyapatite recovered from municipal wastewater using a metal oxide/polymer adsorbent and NaOH desorbing solution. 

The increase of P content with an increase in calcium in the phosphate to calcium ratio indicates that calcium limited the formation of the hydroxyapatite at the lower calcium in the ratio. Limitation of calcium may be because that part of the calcium was used up in the formation of calcium sulphate complex or precipitate. This is a likely possibility because of the high concentration of sulphate in the MBR water and the resin having preference for adsorption of sulphate over phosphate. As in the case of struvite precipitation, the phosphate contents of the hydroxyapatite produced from the desorbed solution collected during the last 30 min were smaller than those produced from the first 30 min desorbed solutions. 

The amount of phosphate adsorbed by the column used in the study was 746 mg (breakthrough capacity of 13.3 mg/g × weight of resin in column of 56 g). The treated volume of water was 78.8 L. Given that the Gordon plant produces 110 ML/year, it is therefore possible that the amount of phosphate recoverable from this plant as phosphate fertiliser is 1041 kg/year. The Gordon plant is a relatively small one compared to other plants in Sydney and therefore the amount of phosphate that can be recovered is also small. At the average rate of phosphate applied to pasture in Australia, *i.e.*, 20 kg/ha/year [31] the amount of phosphate fertiliser able to be produced even with this small plant can meet the phosphate requirement of 50 ha of pasture land.

Regeneration and reusability of the adsorbent was not studied. However, a recent study on the use of this same adsorbent for nitrate removal has shown that the adsorbent can be reused for two more adsorption/desorption cycles without significantly losing its adsorption capacity [32]. The desorption in that study was conducted using 1 M KCl which is stronger than the 0.1 M NaCl concentration used in the current study. Considering this, it is expected that the adsorbent can be satisfactorily reused for few more cycles for phosphate removal as well. However, this suggestion needs to be tested in future studies. 

## 4. Conclusions 

Batch and fixed-bed column experimental results showed that Dowex 21K XLT is a potential adsorbent for removing phosphate from MBR treated water. The Langmuir maximum adsorption capacity of phosphate was 38.6 mg/g. These adsorption capacities are higher than that of nitrate but lower than that of sulphate. The adsorbed phosphate was quantitatively desorbed by leaching the column with 0.1 M NaCl solution. The desorbed phosphate was recovered as struvite when ammonium and magnesium were added at the molar ratio of phosphate, ammonium and magnesium of 1:1:1 at pH 9.5. Phosphate was also recovered from the desorbed solution as hydroxyapatite precipitate by adding calcium hydroxide to the solution at phosphate to calcium molar ratios of 1: 2 and 1:0.5 at pH 7.0. At a lower ratio of 1:0.5, the precipitate contained less P. The P contents of struvite and hydroxyapatite produced were close to those of the commercial phosphorus fertilisers. The study showed there is much potential in recovering P from wastewaters and producing phosphate fertilisers. Doing so will partly overcome the future scarcity of P resulting from the exhaustion of natural phosphate rock reserves.

## Figures and Tables

**Table 1:** Characteristics of MBR treated water.

Parameters	Unit	MBR Treated Water
Phosphate	mg/L	20.0, 35.0 *
Nitrate	mg/L	26.0
Sulphate	mg/L	33.0
TOC	mg/L	8.9
pH		7.0–7.5

* Water containing 20.0 mg/L of phosphate was used for column experiments and 35 mg/L used for batch experiments.

**Table 2:** Chemical composition of the recovered struvite precipitate (mean ± standard error).

Desorption Time (min)	Phosphate Concentration of Desorbed Solution (M)	Chemical Composition, Weight (%)		
		Phosphate	Ammonium	Magnesium
30	0.007	41.7 ± 0.29	10.9 ± 0.68	12.6 ± 0.55
60	0.004	21.7 ± 0.49	7.9 ± 0.68	8.6 ± 0.55

**Table 3:** Chemical composition of the recovered hydroxyapatite precipitate (mean ± standard error).

Desorption Time (min)	Phosphate Concentration of Desorbed Solution (M)	Molar Ratio Phosphate: Calcium	Chemical Composition, Weight (%)	
			Phosphate	Calcium
30	0.007	1.0:0.5	25.1 ± 0.28	35.1 ± 0.27
		1.0:2.0	38.5 ± 0.28	32.8 ± 0.45
60	0.004	1.0:0.5	26.4 ± 0.19	28.4 ± 0.49
		1.0:2.0	29.9 ± 0.58	33.7 ± 0.75
